# Molecular dynamics analysis of the aggregation propensity of polyglutamine segments

**DOI:** 10.1371/journal.pone.0178333

**Published:** 2017-05-25

**Authors:** Jingran Wen, Daniel R. Scoles, Julio C. Facelli

**Affiliations:** 1 Department of Biomedical Informatics, University of Utah, Salt Lake City, Utah, United States of America; 2 Department of Neurology, University of Utah, Salt Lake City, Utah, United States of America; Universidade Nova de Lisboa Instituto de Tecnologia Quimica e Biologica, PORTUGAL

## Abstract

Protein misfolding and aggregation is a pathogenic feature shared among at least ten polyglutamine (polyQ) neurodegenerative diseases. While solvent-solution interaction is a key factor driving protein folding and aggregation, the solvation properties of expanded polyQ tracts are not well understood. By using GPU-enabled all-atom molecular dynamics simulations of polyQ monomers in an explicit solvent environment, this study shows that solvent-polyQ interaction propensity decreases as the lengths of polyQ tract increases. This study finds a predominance in long-distance interactions between residues far apart in polyQ sequences with longer polyQ segments, that leads to significant conformational differences. This study also indicates that large loops, comprised of parallel β-structures, appear in long polyQ tracts and present new aggregation building blocks with aggregation driven by long-distance intra-polyQ interactions. Finally, consistent with previous observations using coarse-grain simulations, this study demonstrates that there is a gain in the aggregation propensity with increased polyQ length, and that this gain is correlated with decreasing ability of solvent-polyQ interaction. These results suggest the modulation of solvent-polyQ interactions as a possible therapeutic strategy for treating polyQ diseases.

## Introduction

The polyglutamine (polyQ) diseases are caused by unstable expansions of CAG repeats resulting in proteins with expanded polyQ tracts. The polyQ diseases include Huntington’s disease (HD), the spinocerebellar ataxias (SCAs 1, 2, 3, 6, 8, 7, 17), dentatorubral-pallidoluysian atrophy (DRPLA), and spinal and bulbar muscular atrophy (SBMA) [1–6]. Pathogenesis in these diseases is associated with abnormal polyQ protein folding [7–9] and resultant neuronal inclusion body formation [8, 10–14]. While polyQ protein folding, stability, and aggregation have been well described for the polyQ diseases [15, 16], the molecular mechanisms leading to protein misfolding and aggregation, at the atomic level, are not well understood.

Computational simulations, using a variety of approaches including Molecular Dynamics (MD), Replica Exchange MD (REMD) and Coarse Grain (CG) have been used to study polyQ segment aggregation in several publications [17] [18] [19] [20] [21] [22] [23] [24] [25] [26] [27] [28] [29] [30]. The overall picture that emerges from these studies, which include both monomers and dimers of different polyQ lengths, is that polyQ segments tend to favor β-sheet structures, which are propense to aggregation. Also, the results indicate that solvent-polyQ interactions can modulate the aggregation ability of polyQ segments. However, there are no consistent studies analyzing the changes of solvent-polyQ interactions as the length of the polyQ segment increases. Therefore, it appears of interest to consistently study how the tendency to aggreagtion is modulated by solvent-polyQ interactionsas the lengh of the polyQ segment increases. These studies would require the simulation of polyQ segments of increasing lengths using the same simulation protocols and explicitly including the polyQ-solvent interactions.

Here we report the study of solvent effects on solution properties, folding, and aggregation propensity of simple polyQ sequences of increasing lengths, as approximate models for the study of solvent effects on the aggregation propensity of polyQ disease related proteins [31].

## Methods

The polyQ tract is the only common region observed in the otherwise very dissimilar polyQ proteins which are associated with polyglutamine diseases, and in all cases the polyQ expansion causes the disease. The threshold length of the polyQ segment that triggers these diseases is around 35 to 40 residues, except in SCA6 which has a shorter threshold of around 19 repeats [32–34]. Therefore, it is of interest to study the solvation behavior of polyQ segments shorter than 20 and longer than 40 repeats to find common features on how solvent interactions may affect the folding of such diverse set of proteins.

We performed MD simulations for polyQ monomers with 18 repeats (Q18), 46 repeats (Q46) and 32 repeats (Q32). These correspond to lengths below the lowest known disease threshold, above the highest known normal threshold and the average repeat length of these two, respectively. A randomly selected extended structure of polyQ was used as the starting structure of the MD simulations. In order to avoid complications due to charged termini [35], the polyQ sequences were capped with an acetyl group in the N-terminus and a N-methylamide group in the C-terminus, i.e. the structures considered here are [acetyl-(Gln)_n_-N-methylamide], where n = 18, 32, and 46 denotes the number of glutamines. xLEaP [36] was used to build the initial configurations, and the Amber force field, AMBER ff99SB [37], was used with a TIP3P water box to provide an explicit simulation of the solvent. A local minimization of the polyQ monomers was done in vacuum before the water box was added. The TIP3P water was included in a truncated octahedral box added to the polyQ monomer with a buffering distance of 9.0 Å between the edges of the box and the polyQ monomer. A second minimization was performed on the solvated system using a non-bonded cutoff distance of 9 Å to minimize the energy of the whole system. The whole system was then heated from 0 K to 310 K and equilibrated for 50 ps, followed by molecular dynamics simulations for 105 ns at the temperature of 310K and constant pressure of 1 atm. The temperature was maintained through the Berendsen thermostat with a coupling time of 0.1 ps. Isotropic position scaling was used to maintain the pressure and a relaxation time of 1 ps was used. The integration time step was 2 fs, and results were recorded every 1 ps.

For each polyQ monomer six independent runs, using different randomly selected initial structures and different random seeds for its initialization, were performed and the results presented here are the average for these six runs. This procedure was adopted to increase sampling of the conformational space, while keeping a manageable MD simulation time. All the MD simulations were done using the Amber 14 molecular simulation package [38] that supports a GPU accelerated PMEMD module, which implements the Particle Mesh Ewald (PME) method for electrostatics [39]. All calculations were performed using the clusters at the Center for High Performance Computing (CHPC) at the University of Utah. Each computing node in the cluster has two Nvidia 2090 GPUs and 12 Intel Xeon (Westmere X5660) processors. After a preliminary study to optimize the efficiency of the GPU-accelerated computing nodes (results not shown), we performed one simulation per GPU to obtain the best throughput performance with the settings of our cluster.

The Cpptraj utility in the Amber 14 tool box [38] was used for most of the analysis. The MD trajectories were re-imaged back to the primary box, and to speed up the analysis, only 1/100 of the frames were processed that is 100 ps per frame in the new trajectory. The secondary structure, hydrogen bond, solvent bridge, radius of gyration, and solvent surface area were calculated using Cpptraj for each simulation trajectory. The Rg value of the polyQ segments was calculated for each frame of the the last 80 ns and used to calculate the exponent factor b in the Rg ~ N^b^ equation. To calculate the exponent factor b, the log transform was done on each data point and a linear regression was used to get the value of b, which corresponds to the slope of the linear regresion.

For each polyQ length, the results of the six independent simulations were averaged, such that all values reported here represent the average values over these six runs. Only the last 80 ns of the MD trajectories were considered to avoid transient effects (see S4 Fig). The Pearson's product moment correlation, also known as r, was used to measure the strength and direction of any linear correlation between the two interested variables presented here and the p value was used to test significance. Statistical analyses were performed using R [40], figures were plotted with ggplot2 package [41] and Gnuplot [42], and VMD was used for trajectory visualization [43].

## Results

### Overall GPU performance

The systems considered here, including both polyQ monomer and water solvent (Table 1), are large enough to exhibit excellent scaling when using GPUs. The GPU version of the Amber PMEMD module on the GPU furnished nodes provides highly consistent speedups, with an average factor of 8.5 times speedup over the CPU times.

**Table 1:** Comparison of AMBER CPU and GPU performance for simulations of polyQ monomers in explicit solvent with different number of repeats.

	number of atoms	CPU Performance (ns/day)	GPU performance (ns/day)
Q46	341,249	0.30±0.01	2.73±0.02
Q32	134,359	0.86±0.00	7.49±0.04
Q18	34,407	3.55±0.01	27.75±0.12

### Secondary structure

**Fig 1:**
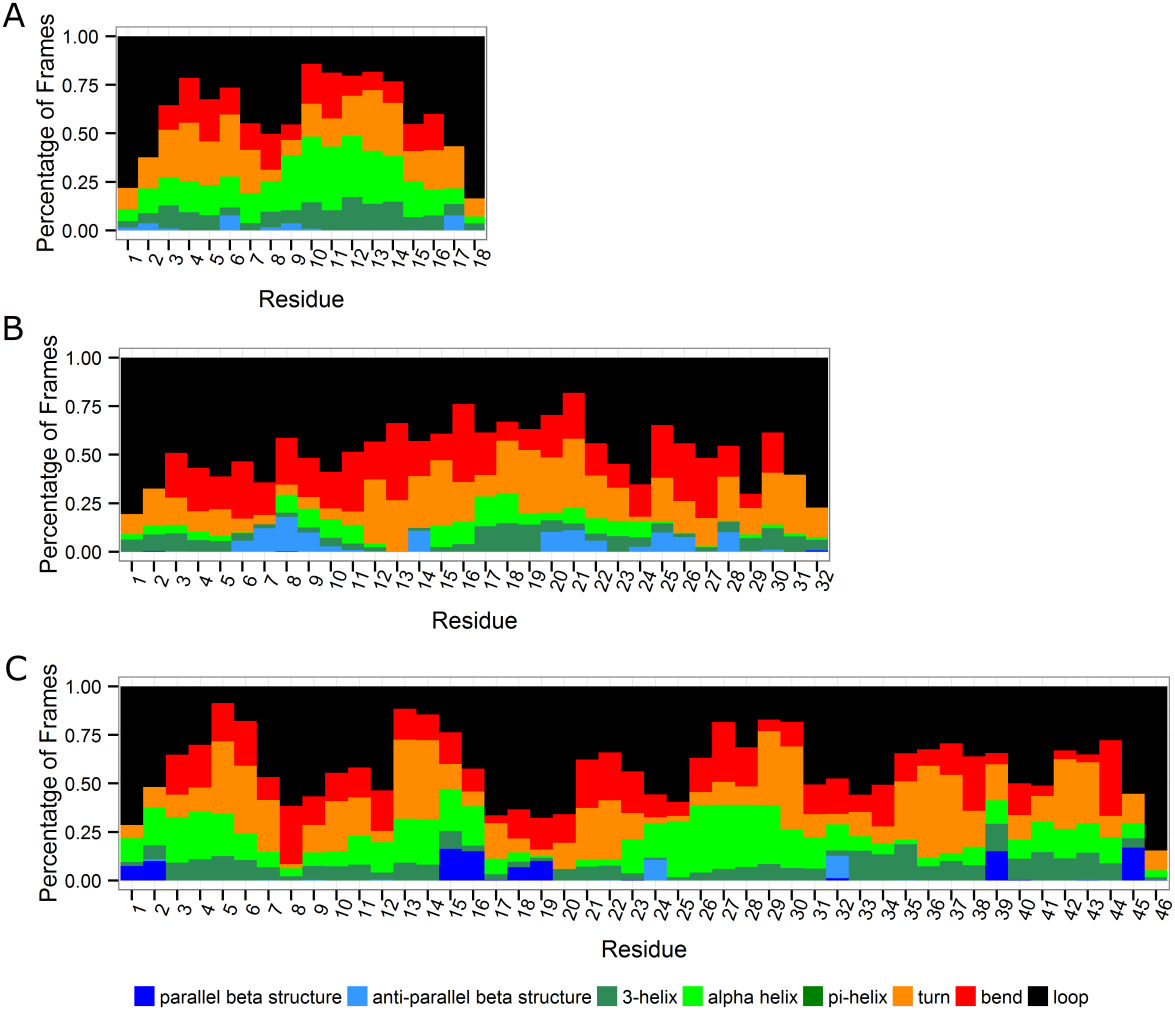
Secondary structure of polyQ fragments of different lengths. **A**. Q18. **B**. Q32. **C**. Q46. Colors indicate secondary structures of different types. Blue: parallel β structure; Sky blue: anti-parallel β structure; Dark green: 3-helix; Green: α-helix; Olive: pi-helix; Orange: turn; Red: bend; Black: loop. X-axis: residue index; Y-axis: percentage of frames in the 80 ns simulations. These results are the averaged ones over the six runs performed here.

**Fig 2:**
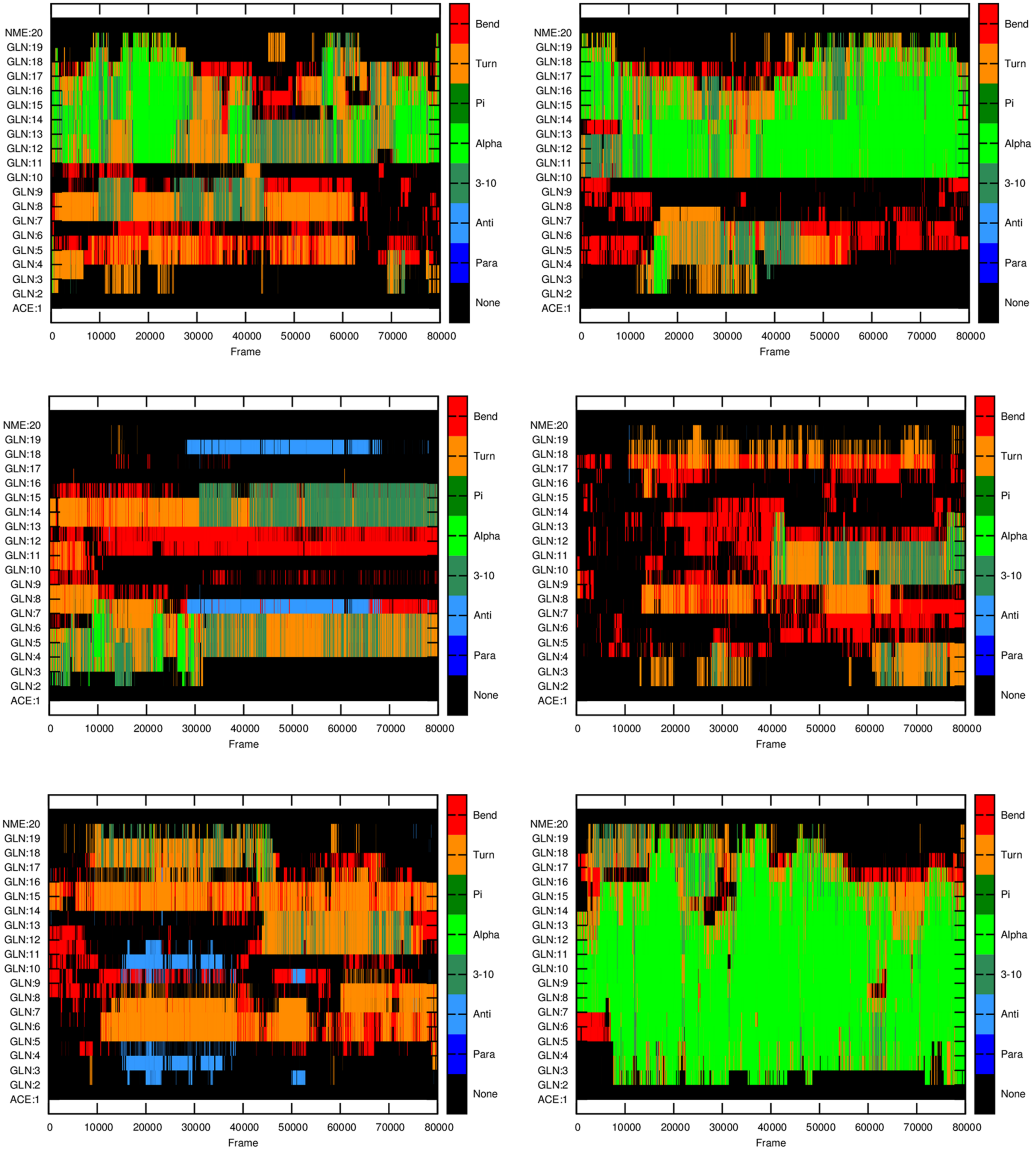
Secondary structure of Q18 monomers at different time frames for each of the six independent MD runs performed. X-axis: frame index with each frame representing 100 ps of simulation; Y-axis: residue index indicating the secondary structure as depicted at the right panel.

**Fig 3:**
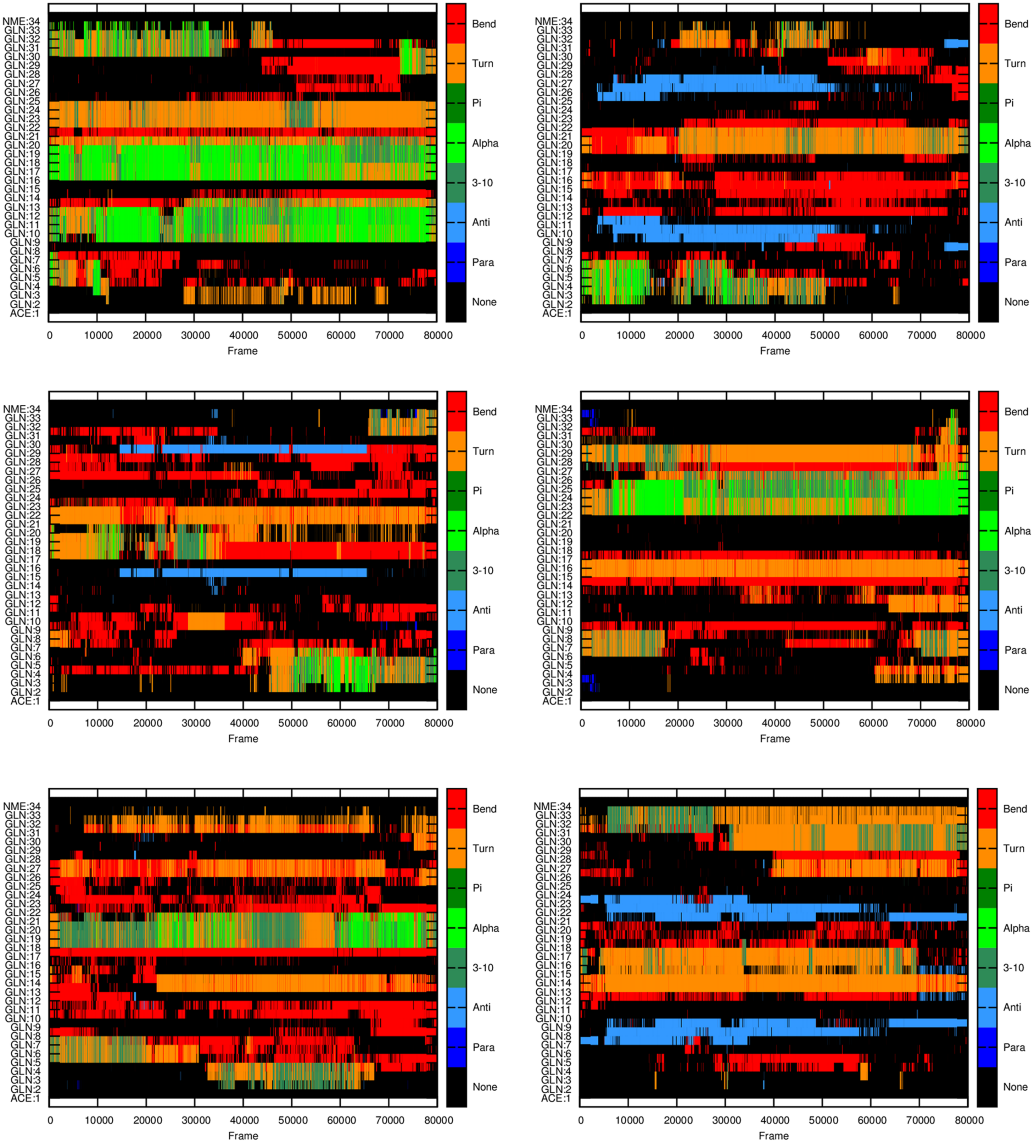
Secondary structure of Q32 monomers at different time frames for each of the six independent MD runs performed. X-axis: frame index with each frame representing 100 ps of simulation; Y-axis: residue index indicating the secondary structure as depicted at the right panel.

**Fig 4:**
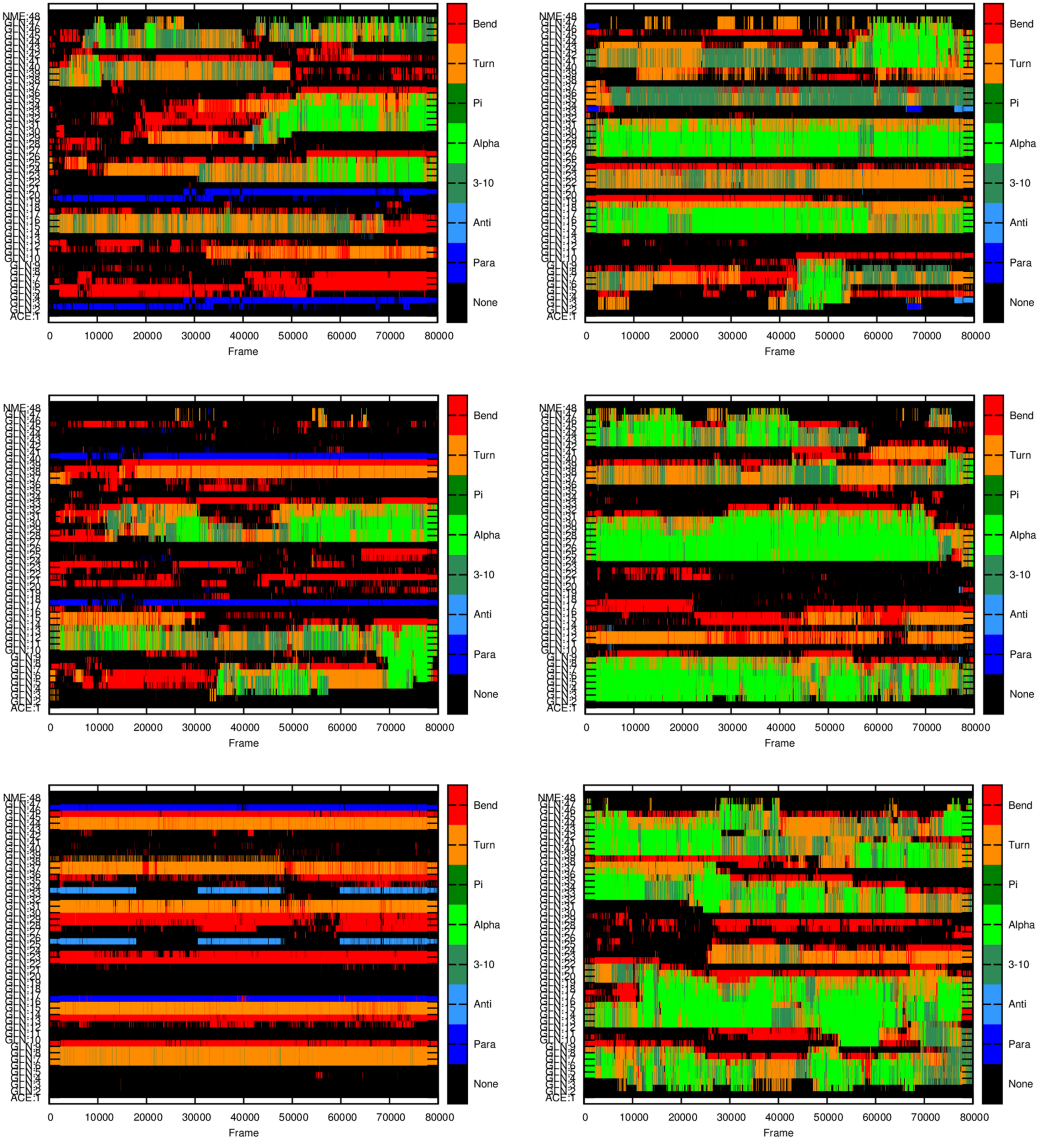
Secondary structure of Q46 monomers at different time frames for each of the six independent MD runs performed. X-axis: frame index with each frame representing 100 ps of simulation; Y-axis: residue index indicating the secondary structure as depicted at the right panel.

Our simulations show that polyQ monomers can adopt various secondary structures instead of fixed structures during the last 80 ns simulations (Fig 1). The distribution of these conformations, changes substantially depending on the initial configuration (Figs 2–4). This is consistent with previous results showing that polyQ monomers are disordered [31]. Q18 monomers show the highest proportion of helical structures including 3-helix and α-helix, whereas Q32 monomers adopt the lowest proportion of helical structures on average. It is apparent from Fig 1 that the number of β-structures, especially parallel ones, increases as the length of the polyQ segment increases. This is an important structural change as it has been established that parallel β-structures are a precursor for initiating aggregation [44].

The stability of the β-structures, as a function of time, is also different for monomers of different lengths (Figs 2–4). The simulations show that the parallel β-structures in Q46 monomers are very stable and most of them can last for the entire simulations (Fig 4), whereas in Q18 and Q32 these structures are less stable, occurring only in 0.1% of the simulation time in Q18 (Fig 2) and around 1% of the time for Q32 (Fig 3).

### Hydrogen bonding

Hydrogen bonding plays a critical role in polyQ folding and stability [45], therefore changes in hydrogen bond (HB) patterns with polyQ expansion may be a signal of changes in folding and aggregation propensity. Of particular interest is the balance between intra-polyQ and polyQ-solvent hydrogen bonds. In this study, we term the hydrogen bond as intra-polyQ if both donor and acceptor are from glutamine residues, and hydrogen bonds between glutamine residues and solvent water molecules are called solvent-polyQ hydrogen bonds. As the amide group in the sidechain of a glutamine can provide one hydrogen donor (hydrogen in NE2) and two hydrogen acceptors (NE2 and OE1), the intra-polyQ sidechain hydrogen bonds can be either backbone-sidechain or sidechain-sidechain. The hydrogen bonds are identified using the hbond command in Cpptraj program in the Amber 14 Toolbox. The distance cut-off is set at 3.5 Å, and the cut-off of angle between the donor-hydrogen-acceptor is set at 120°. Solvent bridges, defined as a solute-solvent-solute interaction, were also identified using the hbond function of Cpptraj with default values. Therefore, there can be more than one water molecule, surrounding one glutamine that meets these criteria. The number of hydrogen bonds reported in this study represents the dynamic count of the number of hydrogen bonds detected by Cpptraj over the last 80 ns of all the trajectories generated by the six MD simulations.

#### Intra-polyQ hydrogen bonds

Using the procedure described above, the number of hydrogen bonds is counted for each individual frame in the last 80 ns MD simulation for each MD run. The normalized count of hydrogen bonds per 100 Qs, which is the number of hydrogen bonds normalized by the length of the polyQ segment multiplied by 100, is calculated as a measure of the relative ability of polyQ monomers to form hydrogen bonds.

**Fig 5:**
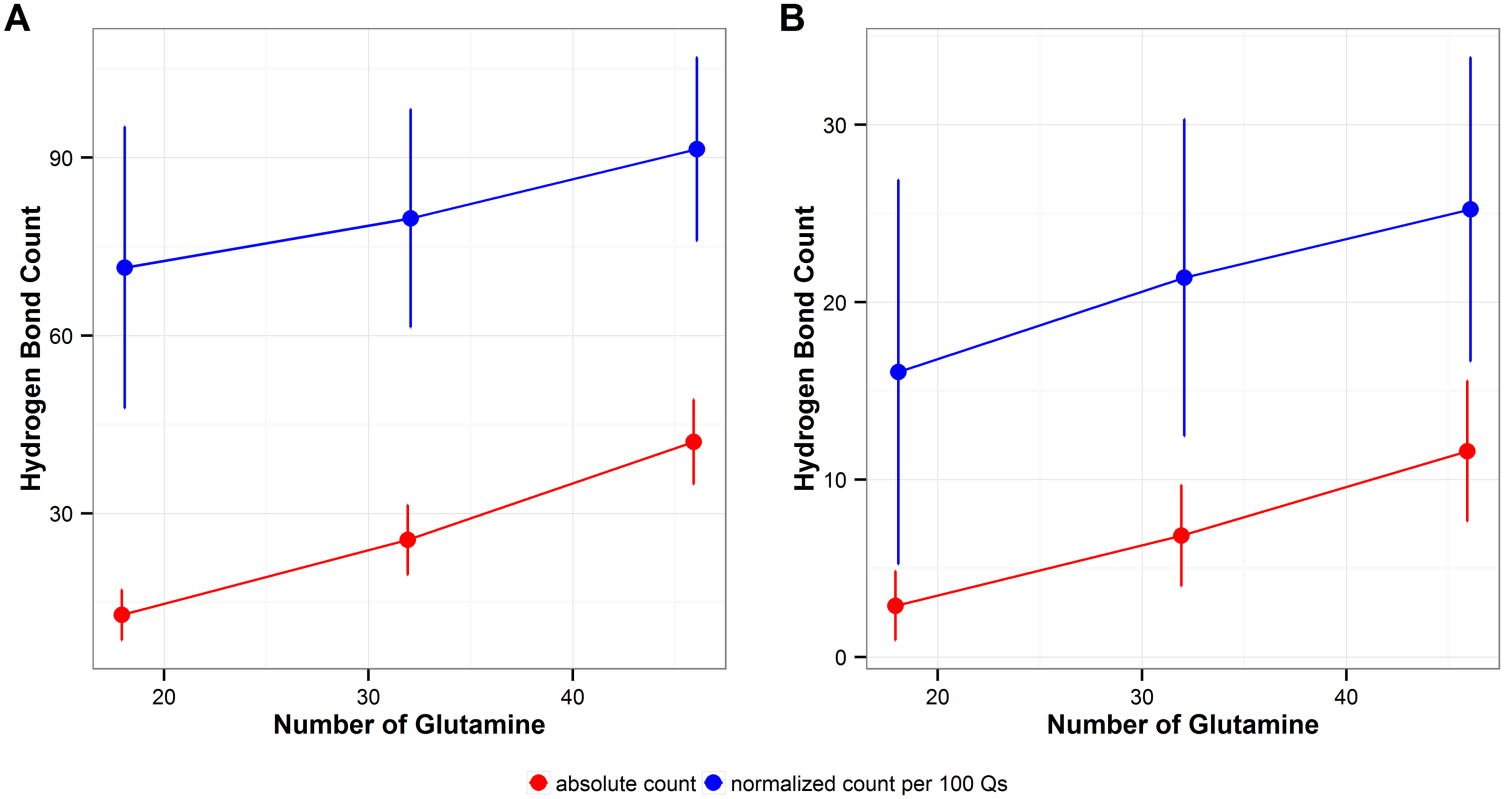
Intra-polyQ hydrogen bonding. **A**. Total number of intra-polyQ hydrogen bonds. **B**. Sidechain-sidechain hydrogen bonds. Red: total count of hydrogen bonds, Blue: normalized count of hydrogen bonds per 100Qs. The error bars represent the standard deviation of the average values calculated over the six independent MD runs performed in this study.

As expected longer polyQ monomers adopt more intra-polyQ hydrogen bonds than shorter ones (Fig 5A, red), but the normalized count of intra-polyQ hydrogen bonds per 100 Qs also increases as the monomer length increases (Fig 5A, blue), indicating a higher propensity to form intra polyQ HB as the length of the segment increases.

When considering intra-polyQ hydrogen bonds formed with the glutamine sidechains, the number of sidechain-sidechain hydrogen bonds increases with the number of glutamines in the polyQ tract (p<0.001, r = 0.84) (Fig 5B, red). For polyQ monomers of the same length, the number of sidechain-sidechain hydrogen bonds is consistent among simulations, and independent of the secondary structure (S1 Fig). When normalized by the number of glutamines in the polyQ tract, the normalized count of sidechain-sidechain hydrogen bonds per 100Qs also increases with polyQ length (p<0.001, r = 0.39) (Fig 5B, blue). The normalized count of hydrogen bonds per 100 Qs formed by glutamine sidechains, including both sidechain-sidechain and sidechain-backbone hydrogen bonds, are similar in 32 Q and 46Q polyQs, but are fewer in the 18Q polyQ (S2 Fig).

**Fig 6:**
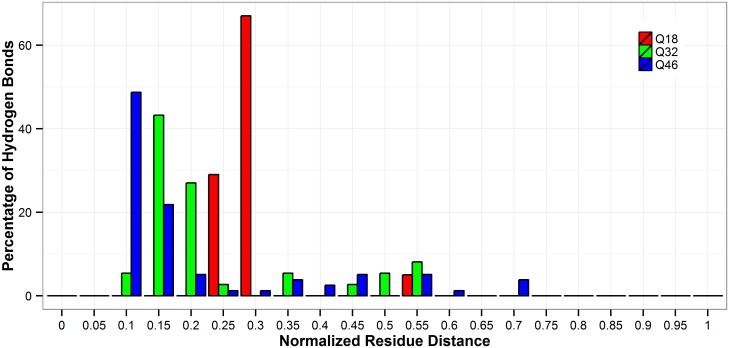
Distance distribution of observed hydrogen bonds with more than 50% frequency. The normalized distance is calculated as (|acceptor residue index-donor residue index|+1)/(the number of repeat in polyQ). Red: Q18; Green: Q32; Blue: Q46.

At the residue level, all polyQ tracts studied here show some common hydrogen bond patterns. The results of this study show that, in all of the repeat lengths studied here, the i^th^ residue prefers forming hydrogen bonds with residues in the positon of i+2, i+3 or i+4 (S3 Fig). We verified that both backbone-backbone and sidechain hydrogen bonds contributed to the patterns of i+2, i+3, and i+4, but that the backbone-backbone hydrogen bonds contributed more than sidechain ones. Some hydrogen-bonded residue pairs are ‘hot’ in all the polyQ segments studied here and this trend is independent of the lengths of the polyQ monomers. Residues 1 and 4 show hydrogen bond propensity in 4 out of the 6 MD simulation runs of 18Q, 32Q, and 46Q polyQ segments. In addition to these common patterns, the intra-polyQ hydrogen bonds also have length-dependent features. The long-ranged hydrogen bonds considered here are the ones formed by two glutamines with a sequence distance longer than at least half of the length of the polyQ monomer. The percentage of long-ranged hydrogen bonds is greater in the longer polyQ tracts than that shown in the shorter ones. For example, when considering hydrogen bonds with a time frequency greater than 50%, 5% of the hydrogen bonds in Q18 are long-ranged ones (Fig 6), whereas 8.1% and 10.3% of the hydrogen bonds are long-ranged ones in Q32 and Q46, respectively. For Q18, a long-lived hydrogen bond can occur between glutamines that are 9 residues apart in the polyQ sequence, and this distance can extend to 15 residue in Q32 and 30 residues in 46Q polyQ monomers.

#### Solvent-polyQ hydrogen bonding

**Fig 7:**
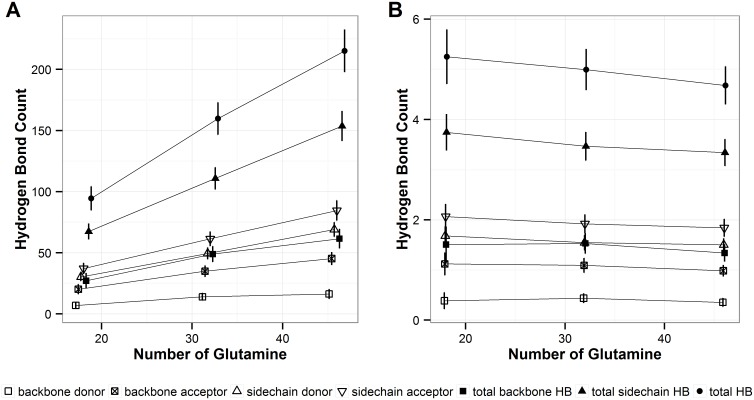
Solvent-polyQ hydrogen bond count. **A**. Total count. **B**. Count normalized by polyQ length. Shapes indicate hydrogen bonds of different types. The error bars represent the standard deviation of the average values calculated over the six independent MD runs performed in this study.

**Fig 8:**
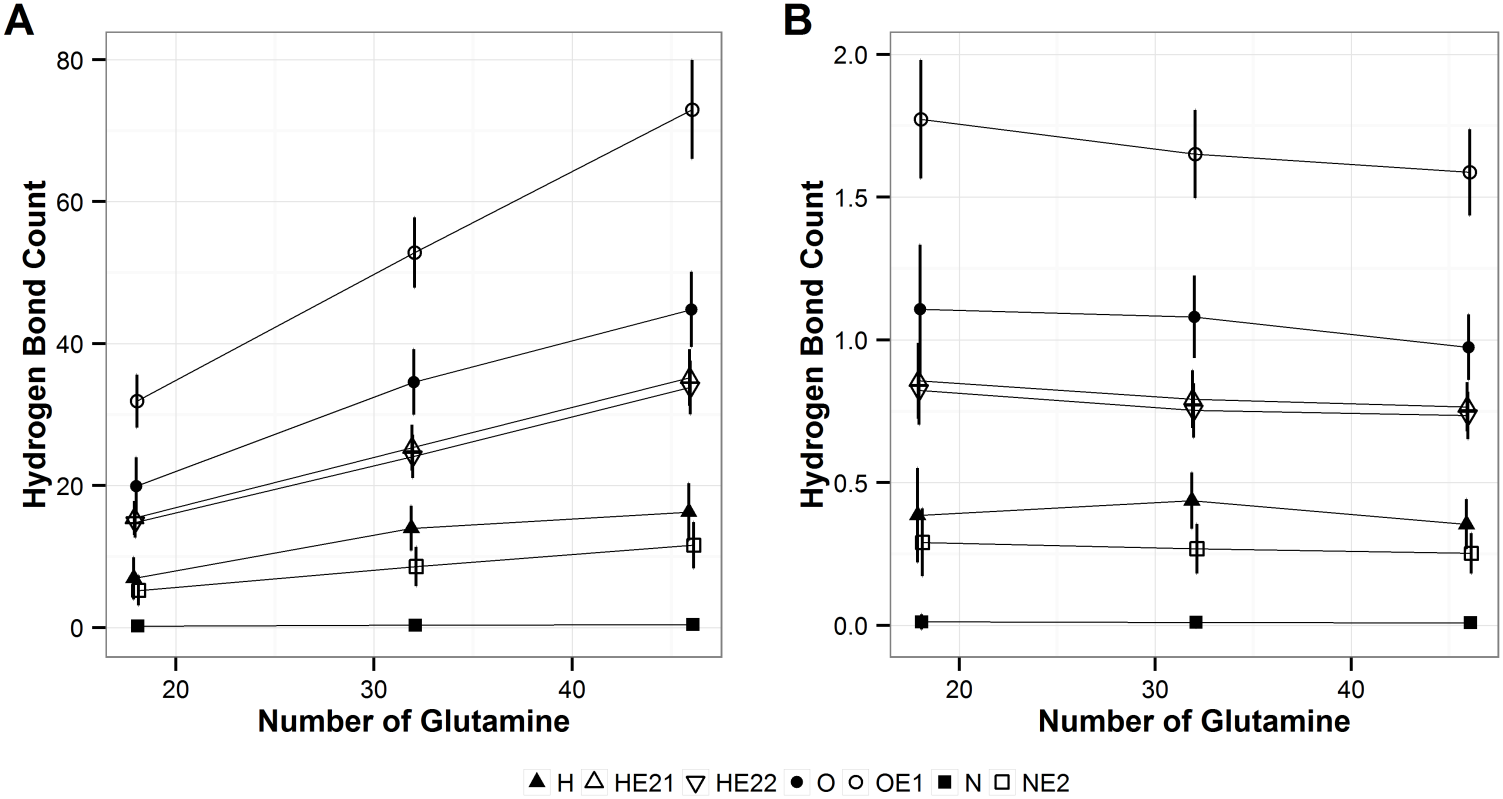
Solvent-polyQ hydrogen bond count at the atomic level. **A**. Total count. **B**. Count normalized by polyQ length. Shapes indicate hydrogen bonds of different donor and acceptor types. The error bars represent the standard deviation of the average values calculated over the six independent MD runs performed in this study.

As expected, the number of solvent-polyQ hydrogen bonds, which are calculated using the criteria defined in the above section, increases as the length of polyQ monomers increases (Fig 7A). The slope of the increase is different among different types of hydrogen bonds, with sidechain solvent hydrogen bonds increasing the greatest (Fig 7A). However, when the total number of intra-polyQ hydrogen bonds is normalized by the number of repeats in the polyQ segment, this normalized number of hydrogen bonds decreases as the polyQ length increases (Fig 7B), which is the reversed trend from what observed for the normalized number of intra-polyQ hydrogen bonds. When classified at the atomic level, the number of hydrogen bonds using each atom, shown in Fig 8, also increases with the length of polyQ (Fig 8A), with sidechain O-mediated hydrogen bonds increasing the greatest. However, when normalized by the polyQ segment length, the number of sidechain O-mediated hydrogen bonds decreases with the polyQ length, as did the backbone O-mediated hydrogen bonds (Fig 8B). The number of normalized hydrogen bonds formed by other atoms do not change substantially, and are similar among polyQs with different lengths (Fig 8B).

#### Intra-polyQ hydrogen bond vs solvent-polyQ hydrogen bond

**Fig 9:**
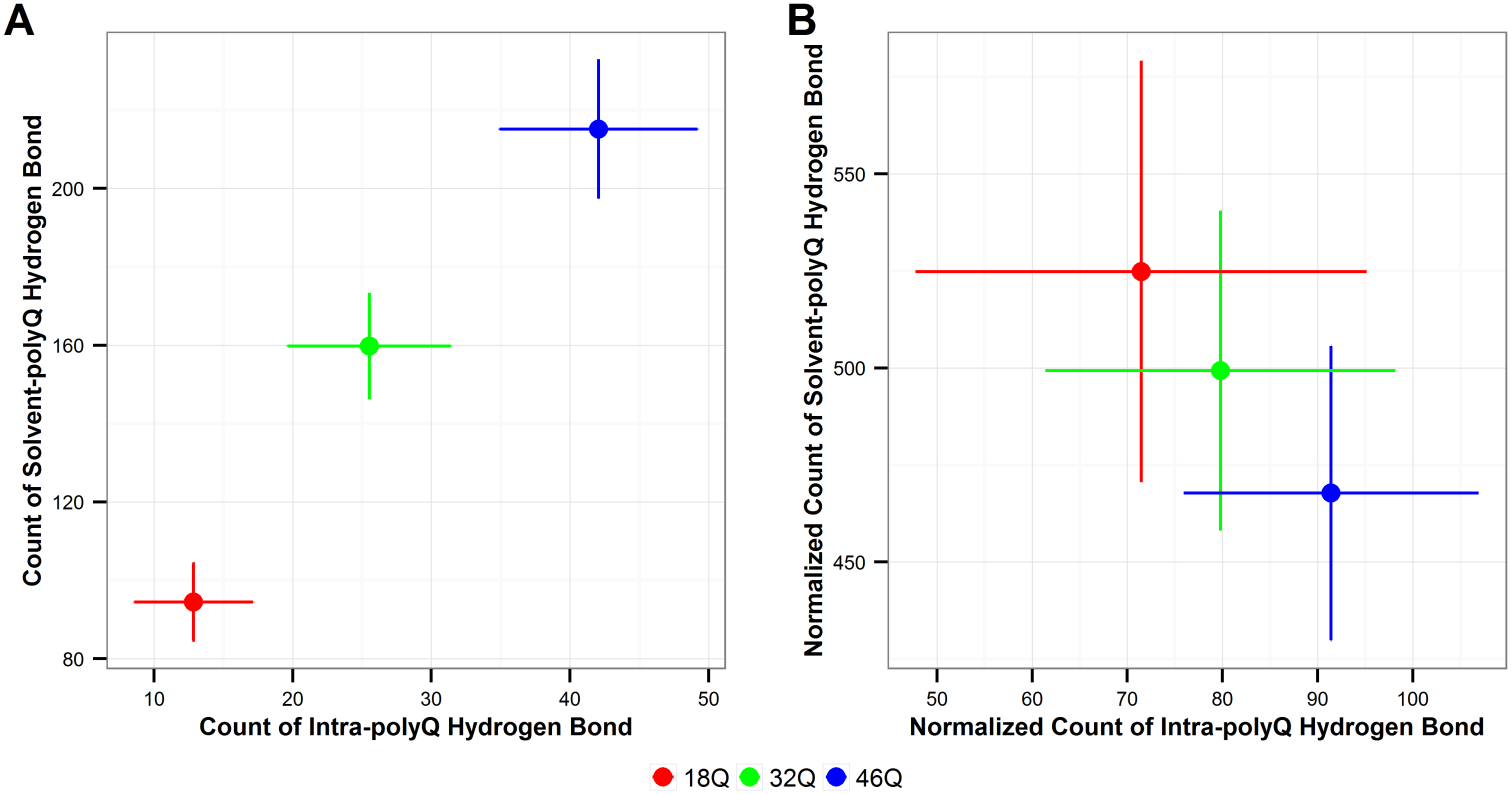
Number of intra-polyQ hydrogen bonds vs. the number of solvent-polyQ hydrogen bond. **A**. Total count. **B**. Normalized count per 100 Qs. The error bars are the standard deviation from all 6 MD simulation runs for each polyQ length. Red: Q18; Green: Q32; Blue: Q46. The error bars represent the standard deviation of the average values calculated over the six independent MD runs performed in this study.

For each simulation time frame, the number of intra-polyQ hydrogen bonds and the number of solvent-polyQ hydrogen bonds are calculated and the values are plotted in Fig 9. As expected, there is strong positive linear correlation between the total number of hydrogen bonds of both types that increases with the length of polyQ tract (Fig 9A), but the correlation changes to a negative relationship (or flat in the worst case scenario given by the error bars) when considering the normalized count per 100 Qs (Fig 9B). This change in the relationship between intra-polyQ and solvent-polyQ hydrogen bonds is a very strong indication that the relative proportion of intra-polyQ hydrogen bonds increases in detriment of solvent-polyQ ones for longer repeat (Fig 9B).

### Solvent bridges

**Fig 10:**
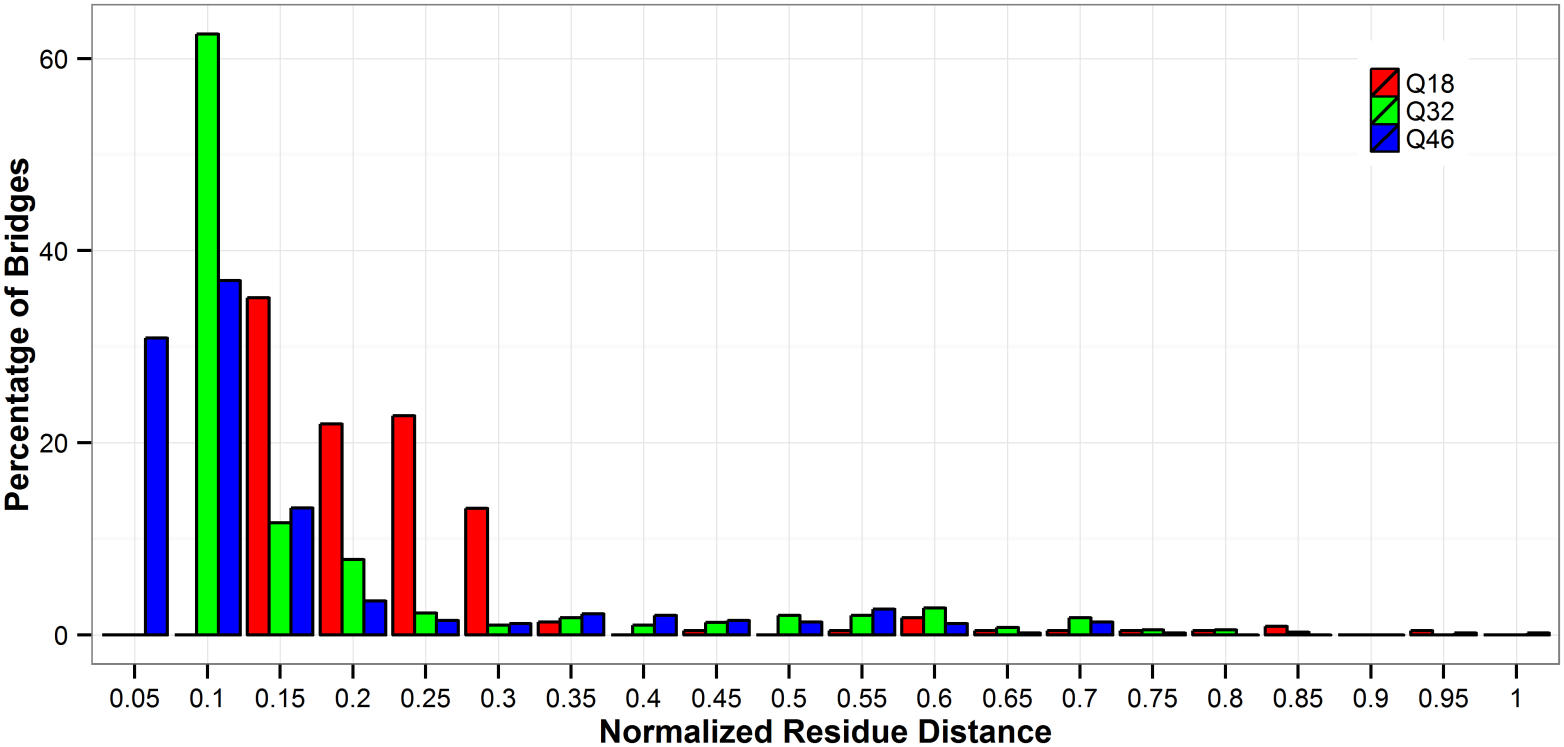
Distance distribution of observed solvent bridges lasting more than 100 frames in the simulation. The normalized distance is calculated as (|acceptor residue index-donor residue index|+1)/(number of repeat in polyQ). Red: Q18; Green: Q32; Blue: Q46.

Water solvent molecules can form bridges with glutamine residues in the polyQ tracts, and these bridges can affect folding and structure stability of polyQ tracts. Therefore, it may be expected that if polyQ tracts with different lengths have different solvent bridge patterns, their folding and structural integrity will also be affected. Fig 10 depicts the frequency of solvent-bridged glutamine pairs with the normalized residue distances. The bridges considered here are the ones that show in more than 100 time frames which corresponds to at least 10% of the simulation time. We find that the frequencies of occurrence, for the observed bridges, range from 1% to 50%. Although the number of long-ranged bridges is small among all three polyQ lengths considered here (Fig 10), polyQs with 32Q and 46Q repeats form more long-ranged bridges than the polyQ monomers with 18Q repeats. 10.6% and 7.2% of these bridges are long-ranged ones in 32Q and 46Q polyQs, whereas only 5.3% of the bridges are in long range in the 18Q polyQs. These results are consistent with the above discussion on the hydrogen bond results, both of which show a substantial decrease of solvent interactions and likely more compact structures as the length of the polyQ tracts increases.

### Radius of gyration (Rg)

The Rg is used to describe the compactness of a protein. It is defined as the root mean square distance of the collection of atoms from their common center of gravity.

**Fig 11:**
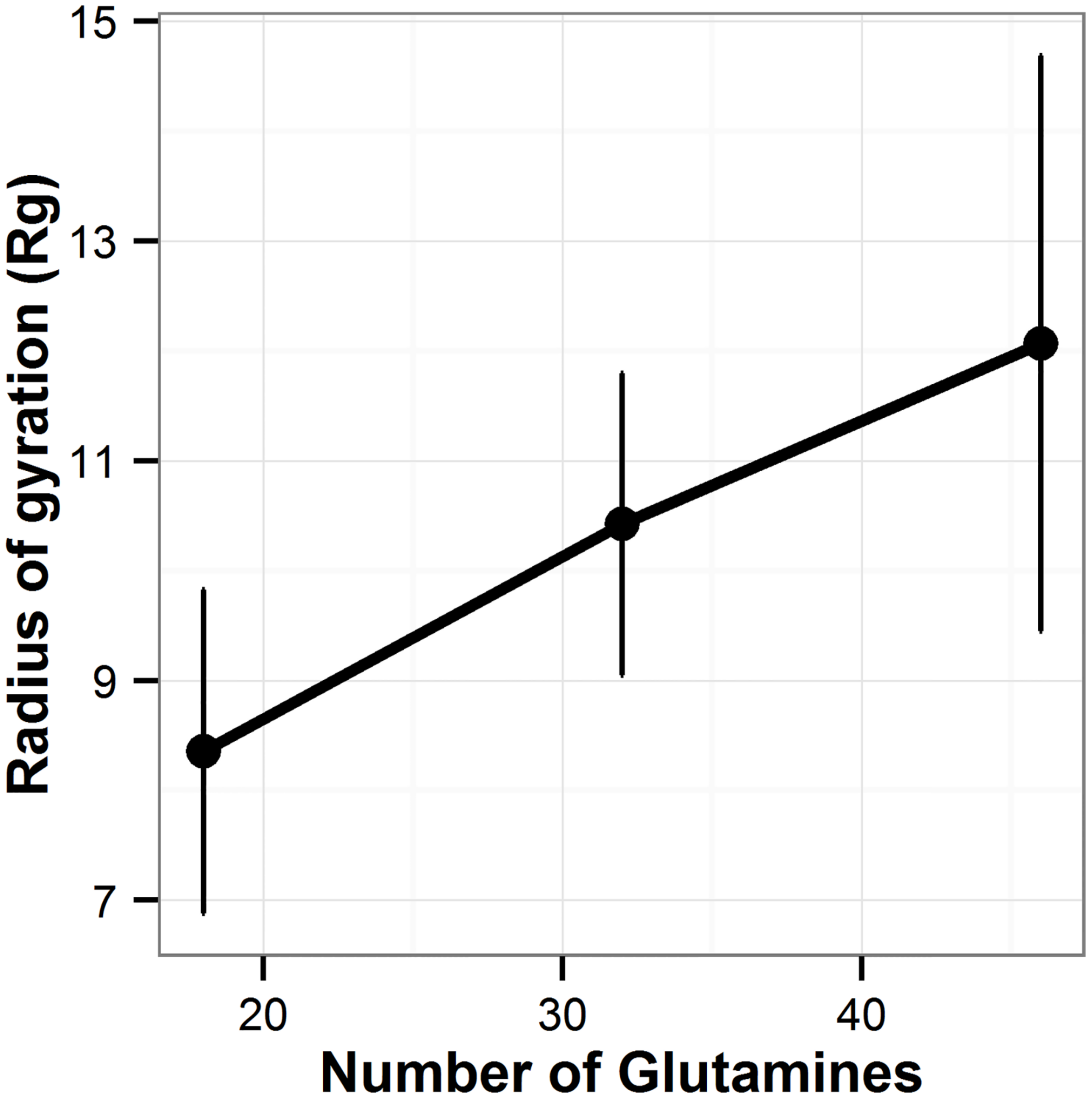
Scaling laws for polyQ monomers in water. Dots represent the Rg values for each polyQ length averaged over the six MD run performed here. The error bars represent the standard deviation of the average values calculated over the six independent MD runs performed in this study.

For flexible polymers, the Rg value is proportional to N^b^, where N is the length of the polymer [46] [47] [48] and b is characteristic of the solubility of the polymer. A good solvent is characterized by an exponent of ~ 0.59, as chain-solvent interactions are preferred, whereas a poor solvent has an exponent value of ~ 0.33, as the chain collapses to minimize contact with solvent [46, 47] as its length increases. Using the last 80 ns simulation data (S4 Fig) we find a value of b = 0.39 ±0.01 for the polyQ segments in water solution studied here (Fig 11). This indicates that, as the length of polyQ segment increases, there is a tendency of preference for polyQ-polyQ interactions to polyQ-solvent interactions. This is consistent with the results of previous sections in this paper. The results of Rg indicates that longer polyQ segments are less soluble, which is also consistent with an increase of their propensity to aggregation as the length of the polyQ increases.

### Solvent accessible surface area (SASA)

**Fig 12:**
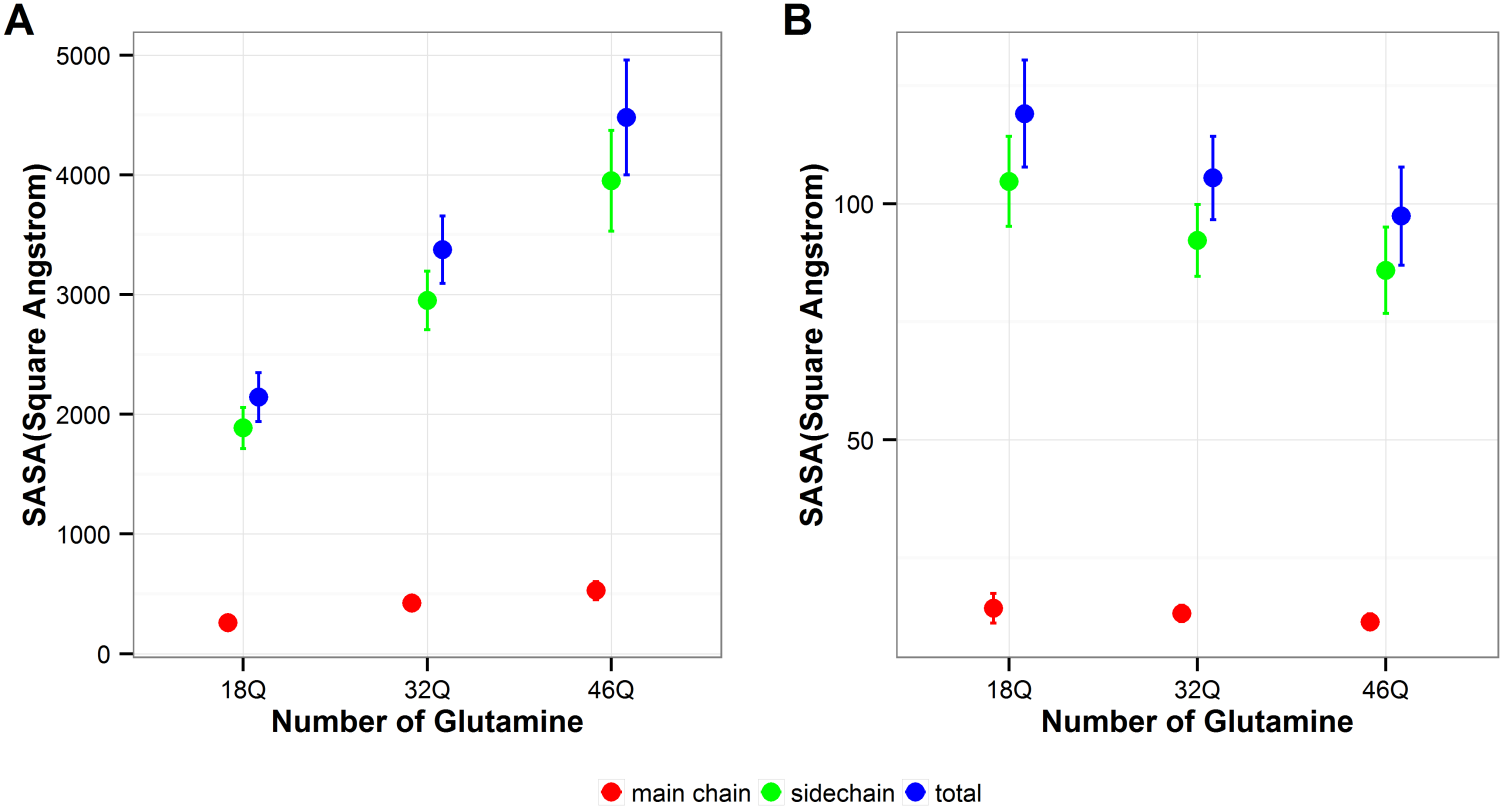
Solvent accessible surface area. **A**. Total SASA. **B**. Normalized SASA. Red: backbone SASA; Green: sidechain SASA; Blue: total SASA. The error bars represent the standard deviation of the average values calculated over the six independent MD runs performed in this study.

As expected, the total SASA of polyQ segments studied here increases as the number of polyQ length increases (Fig 12A). Both total backbone and total sidechain SASA follow the same trend, but sidechain SASA increases faster than the backbone SASA. However, when SASA is normalized by the length of polyQ, this normalized SASA decreases with polyQ length (Fig 12B), which is the same trend from what observed for the Rg values. The normalized backbone SASA for all polyQ sequences studied here is on average smaller than 20 Å^2^, therefore it is likely that the polyQ backbone may be buried inside the structures rather than residing at the surface [49]. These results are also consistent with the results of previous sections, all of which indicate that the ability of polyQ monomers to interact with the solvent decreases as the length of the polyQ sequence increases.

## Discussion

Consistent with the fact that the expansion of polyQ sequence beyond a certain threshold, specific for each polyQ disease, triggers pathogenesis [50], numerous observations have suggested that the polyQ tract by itself may play a central role in the pathogenesis of the ten known polyQ neurodegenerative diseases [13].

In this study, three different lengths of polyQ segments are considered, Q18, Q32, and Q46, to cover normal, intermediate and pathological ranges relevant for most of the polyQ diseases. The MD simulations with explicit solvent presented here show that all polyQ segments mainly form random-coiled structures, which is consistent with previous literature studies [20]. But the results in this study also show an increasing propensity to form helical and β structures as the number of glutamines increases in the polyQ tract. The type of β-structures are different among polyQ monomers of different lengths. The β-structures in Q46 are dominated by parallel β-structures, whereas for Q18 and Q32, the majority are anti-parallel β-structures. While Q18 and Q32 polyQ monomers can form parallel β-structures, these structures are less stable and do not last till the end of the simulations (Figs 2 and 3). On the contrary, for Q46 the parallel β-structures, once formed, can last to the end of the simulations, which may be a clear evidence of the formation of a proto-structure conducive to aggregation.

In this study, the MD simulations of polyQ segments in water predict a b scaling factor for the Rg of 0.39 indicating that water is not a good solvent for polyQ [47, 51]. Consistent with the results of Vitalis *et al* [47], this indicates the decreased preference of solvent-polyQ interaction as the number of repeats increases in polyQ monomers. This observation is also consistent with all the results, obtained here, of the changes in hydrogen bond patterns as the lengths of the polyQ sequences increase.

The results of the normalized SASA also support the idea that the preference of water-polyQ interaction decreases as the length of polyQ increases. Although the total SASA is larger for polyQ monomers with longer repeats, the SASA per residue decreases as the repeat number of polyQ tract increases, especially for the sidechain surface area (Fig 12).

This study also explores the preference of the intra-polyQ vs solvent-polyQ hydrogen bond formation, and the results show that the normalized number of hydrogen bonds per residue increases for the former and decreases for the latter type of hydrogen bond, as the number of repeats increases (Figs 5 and 7). Q18, Q32, and Q46 can potentially form long-ranged hydrogen bonds. Considering the hydrogen bonds that show in more than 50% of the simulation time, in Q18 the great majority of them are short-ranged ones with residues that are only 2- and 3-residue apart. However, long-ranged hydrogen bonds become more common in a larger proportion in Q46 (Fig 6). Driven by the long-distance interaction, it appears that polyQ sequences with longer lengths may be able to fold into more compact structures, which also indicates an increasing propensity to avoid solvent interactions.

All the results presented here consistently point towards an increased propensity to hydrophobicity as the polyQ segments become longer. This raises the hypothesis that the pathogenic cause of the polyglutamine diseases may be rooted in the increased hydrophobicity of their polyQ tracts, which may lead to increasing protein aggregation and causing neural degeneration. While results of this study do not provide direct evidence of the role that the enlargement of polyQ segments play in polyglutamine protein aggregation in the neurodegenerative diseases considered here, given the fact that the only common element of these diseases is the enlargement of polyQ segments in their associated proteins, the results presented here provide impetus to further exploring the hypothesis listed above.

This study is not without limitations. Only polyQ monomers are studied and the inter-molecular interactions among polyQ monomers, which can contribute to aggregation [52], are not included in this study. Additionally, regions flanking the polyQ tract are not considered in this study. Results of our previous study have demonstrated that regions flanking polyQ tracts alter polyQ secondary structure models [53], consistent with findings that these flanking regions alter aggregation of polyQ proteins [28, 54, 55]. However, with the existing study settings, it is easy for us to study the solvation of polyQ tract with the sequence context of the polyQ proteins, both monomers and polymers as well as to explore longer simulation times, in the future.

## Conclusions

This paper studies the effect of solvation on the folding of polyQ segments with repeat lengths in the normal, intermediate, and pathological ranges using molecular dynamics simulations with an explicit water solvent environment. In accordance with the literature, the results of this study show that polyQ monomers can fold, but are disordered. The simulations show that, as the length of a polyQ monomer increases, the water solubility of the polyQ segments decreases, while the propensity to form more compact structures with intra-polyQ hydrogen bonds increases. The results of this study demonstrate gains in aggregation propensity with increased polyQ lengths that correlates with decreasing ability of solvent-polyQ interaction. These results are consistent with previous observations using coarse-grained simulations, and suggest that modulation of solvent-polyQ interaction may be a possible therapeutic strategy for treating polyQ diseases.

## Supporting information

S1 Fig Sidechain hydrogen bonds. Red: sidechain-backbone hydrogen bonds; Blue: sidechain-sidechain hydrogen bonds. Shapes indicate different experiments. From left to right, Q18, Q32 and Q46.
(DOCX)

S2 Fig Number of intra-polyQ hydrogen bonds normalized by the length of polyQ monomer. Green: total number of intra-polyQ hydrogen bonds; Red: backbone-backbone hydrogen bonds; Blue: total sidechain hydrogen bonds; Cyan: sidechain-backbone hydrogen bonds; Sky blue: sidechain-sidechain hydrogen bonds. X-axis: the length of polyQ monomer, Y-axis: number of hydrogen bonds.
(DOCX)

S3 Fig Intra-polyQ hydrogen bonds among different experiments. A, B, and C represent the total intra-polyQ hydrogen bonds; D, E, and F represent the backbone-backbone hydrogen bonds; A and D. Q18. B and E. Q32. C and F. Q46. The data in Figure S3 represents cumulative "Yes" or "No" results, therefore if in one simulation there is at least one hydrogen bond formed between the 2 residues during the last 80 ns simulation, the number is set at 1, and so on. If a HB has been formed in the six independent simulations, the value in the matrix would be 6. Therefore, the values plotted in Figure S3 range from 0 to 6.
(DOCX)

S4 Fig Ratio of gyration calculated at different simulation times for the three polyQ segments studies here. (TIF)

S1 File Excel files containing all data used to construct he figures used in this paper. (ZIP)
