# Supplementary figures and images for: Molecular dynamics analysis of the aggregation propensity of polyglutamine segments
Source: PLoS One. 2017 May 25;12(5):e0178333. doi: 10.1371/journal.pone.0178333 (PMC5444867; doi:10.1371/journal.pone.0178333)

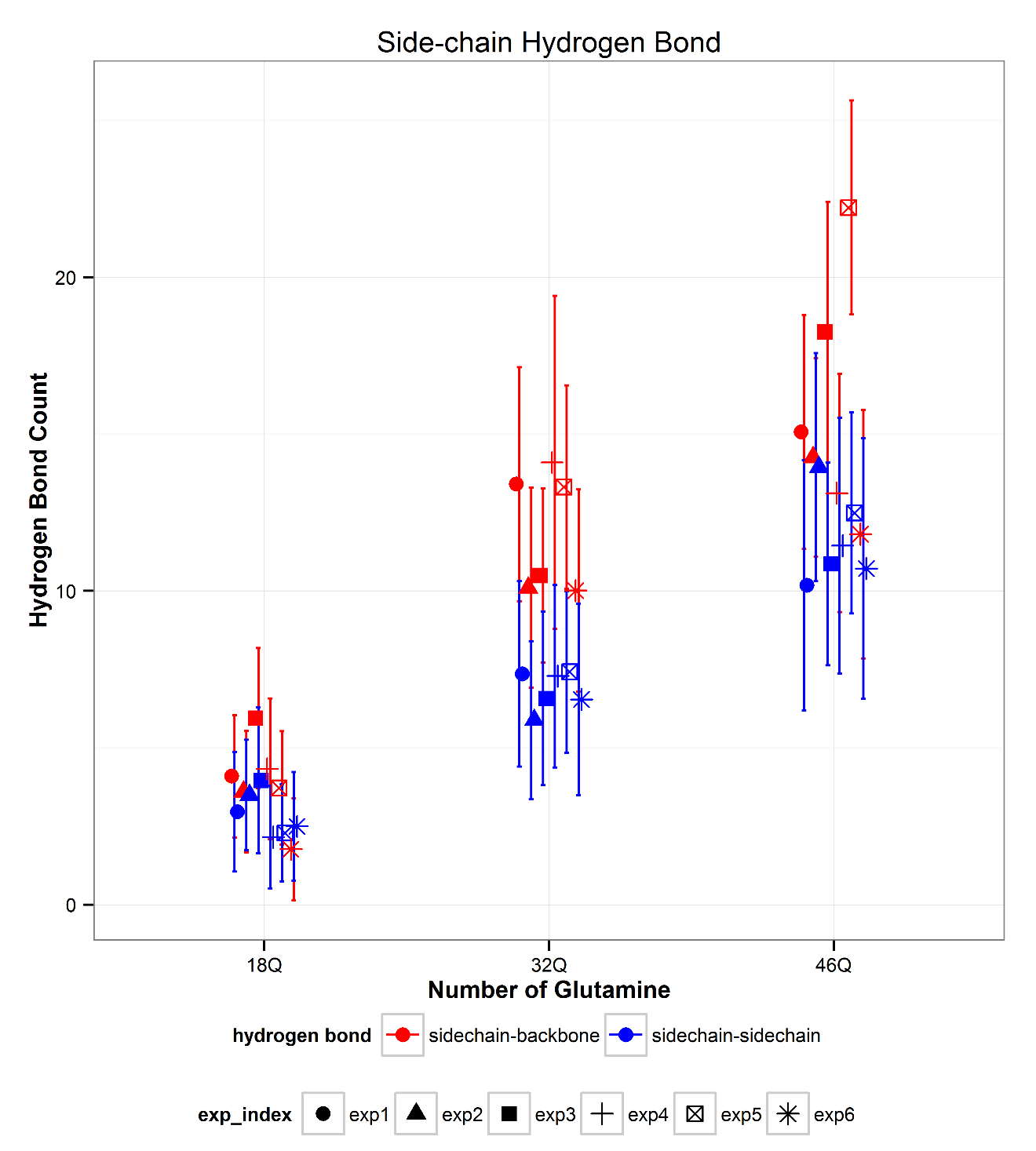

Supplement: S1 Fig — Red: sidechain-backbone hydrogen bonds; Blue: sidechain-sidechain hydrogen bonds. Shapes indicate different experiments. From left to right, Q18, Q32 and Q46. (DOCX) [file pone.0178333.s001.docx]

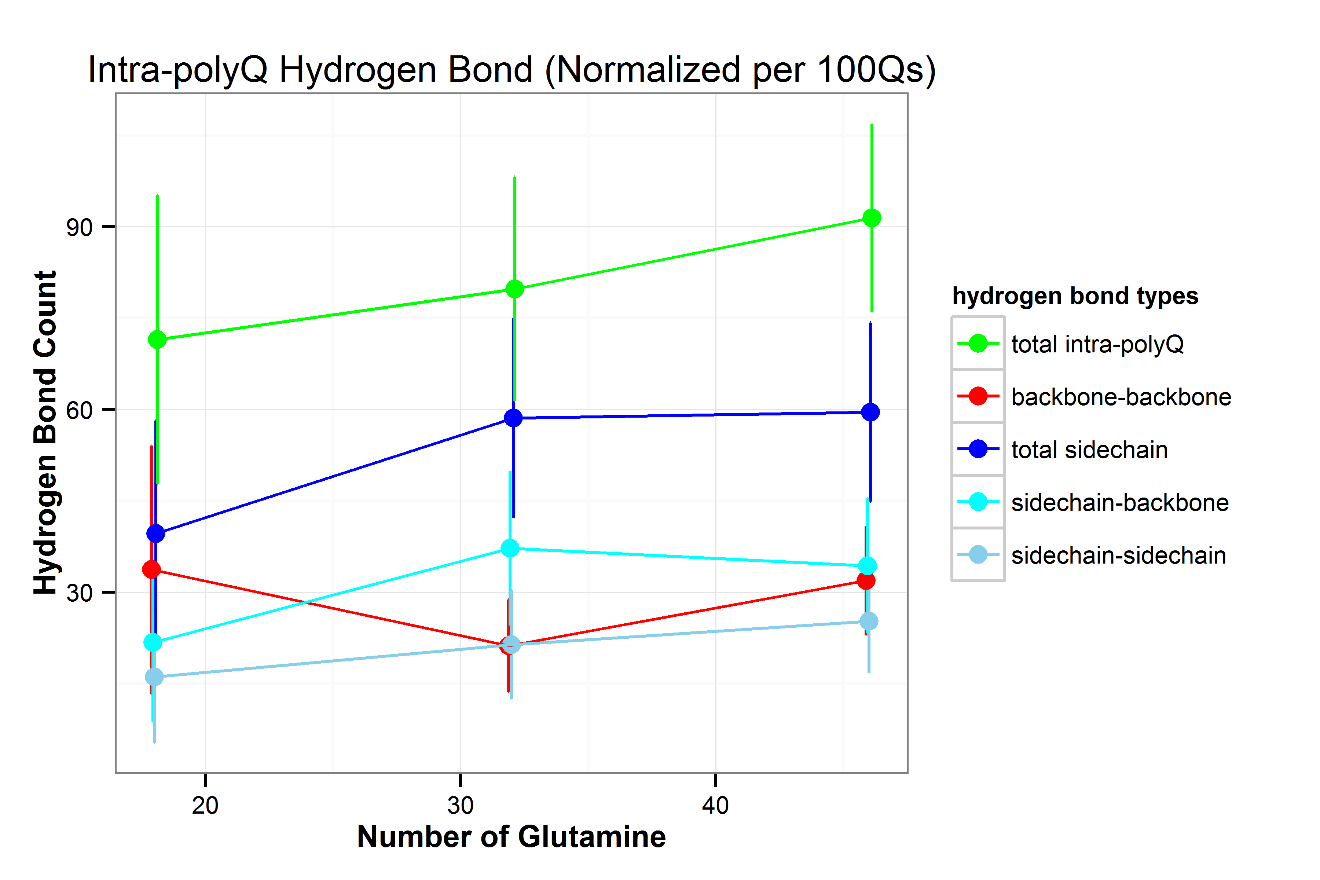

Supplement: S2 Fig — Green: total number of intra-polyQ hydrogen bonds; Red: backbone-backbone hydrogen bonds; Blue: total sidechain hydrogen bonds; Cyan: sidechain-backbone hydrogen bonds; Sky blue: sidechain-sidechain hydrogen bonds. X-axis: the length of polyQ monomer, Y-axis: number of hydrogen bonds. (DOCX) [file pone.0178333.s002.docx]

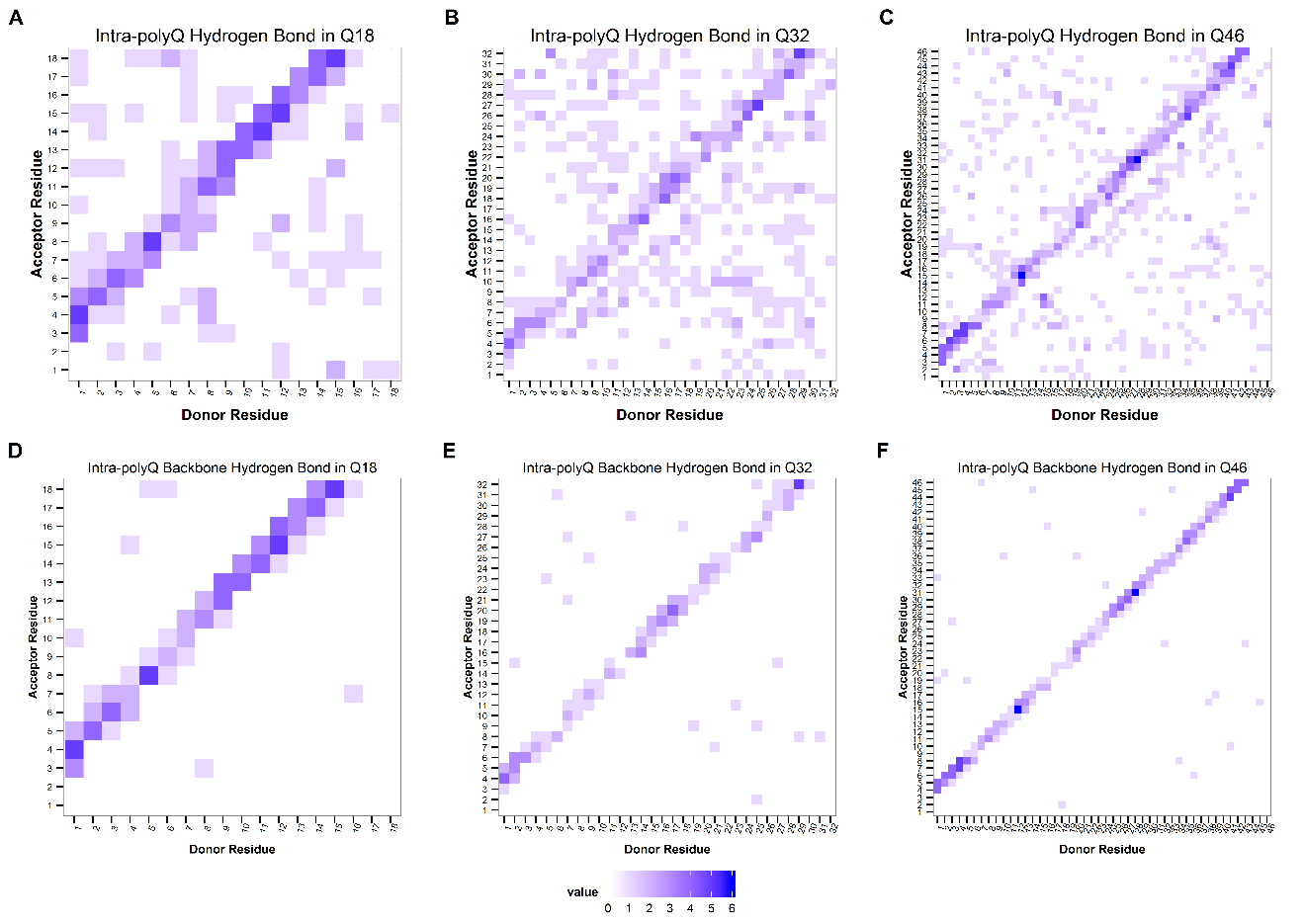

Supplement: S3 Fig — A, B, and C represent the total intra-polyQ hydrogen bonds; D, E, and F represent the backbone-backbone hydrogen bonds; A and D. Q18. B and E. Q32. C and F. Q46. The data in Figure S3 represents cumulative "Yes" or "No" results, therefore if in one simulation there is at least one hydrogen bond formed between the 2 residues during the last 80 ns simulation, the number is set at 1, and so on. If a HB has been formed in the six independent simulations, the value in the matrix would be 6. Therefore, the values plotted in Figure S3 range from 0 to 6. (DOCX) [file pone.0178333.s003.docx]

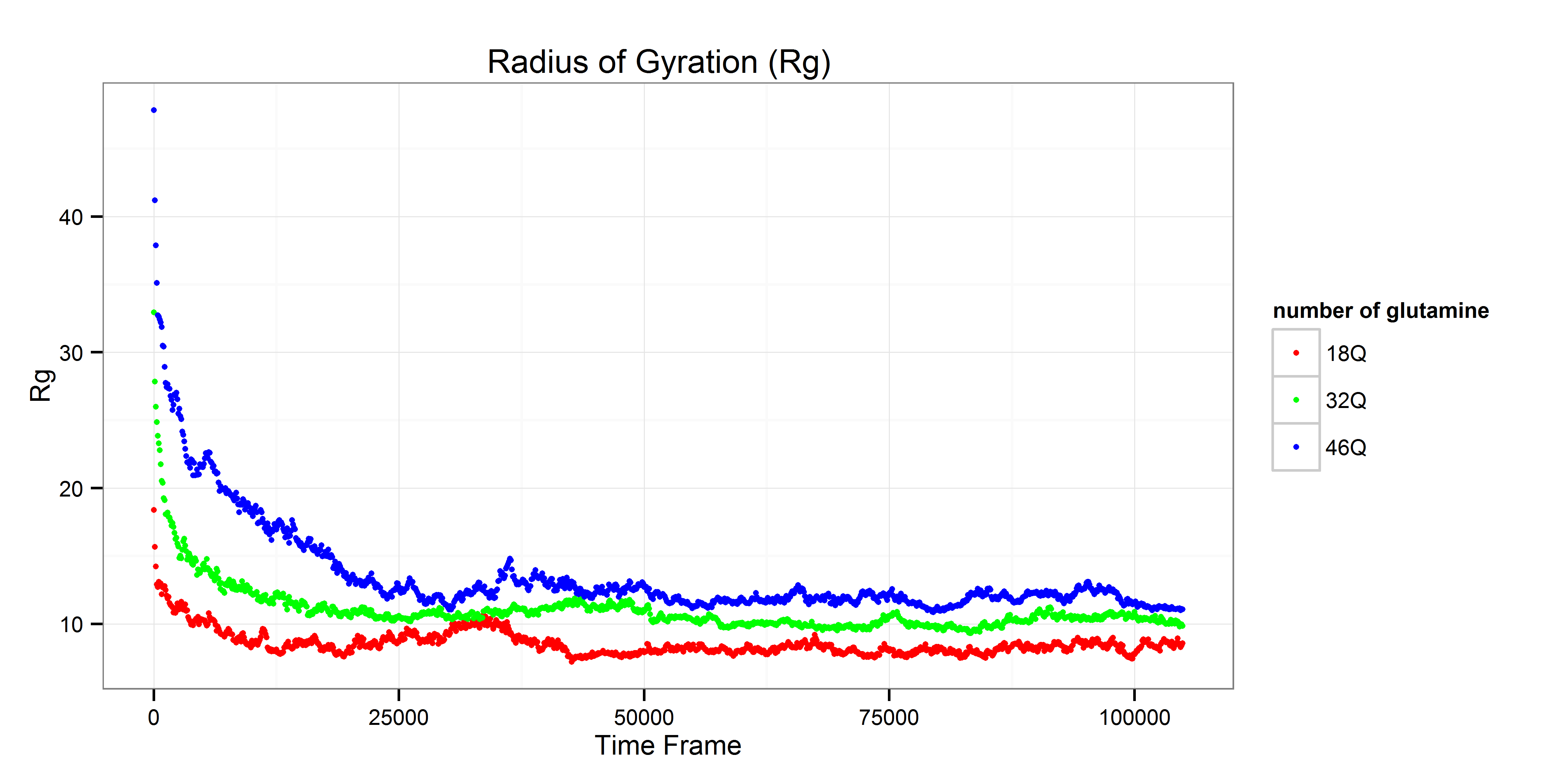

Supplement: S4 Fig — (TIF) [file pone.0178333.s004.tif]
